# Design and Simulation of a Wireless SAW–Pirani Sensor with Extended Range and Sensitivity

**DOI:** 10.3390/s19102421

**Published:** 2019-05-27

**Authors:** Sofia Toto, Pascal Nicolay, Gian Luca Morini, Michael Rapp, Jan G. Korvink, Juergen J. Brandner

**Affiliations:** 1 Institute of Microstructure Technology, Karlsruhe Institute of Technology, Hermann-von-Helmholtz-Platz 1, 76344 Eggenstein-Leopoldshafen, Germany; sofia.toto@kit.edu (S.T.); michael.rapp@kit.edu (M.R.); jan.korvink@kit.edu (J.G.K.); 2 Carinthia Institute for Smart Materials and Manufacturing Technologies (CiSMAT), Carinthia University of Applied Sciences, 9524 Villach/St. Magdalen, Austria; p.nicolay@fh-kaernten.at; 3 Dipartimento di Ingegneria Industriale, Alma Mater Studiorum Università di Bologna, Viale Risorgimento 2, I-40136 Bologna, Italy; gianluca.morini3@unibo.it

**Keywords:** vacuum sensor, Pirani, surface acoustic waves, wireless sensors

## Abstract

Pressure is a critical parameter for a large number of industrial processes. The vacuum industry relies on accurate pressure measurement and control. A new compact wireless vacuum sensor was designed and simulated and is presented in this publication. The sensor combines the Pirani principle and Surface Acoustic Waves, and it extends the vacuum sensed range to between 10^−4^ Pa and 10^5^ Pa all along a complete wireless operation. A thermal analysis was performed based on gas kinetic theory, aiming to optimize the thermal conductivity and the Knudsen regime of the device. Theoretical analysis and simulation allowed designing the structure of the sensor and its dimensions to ensure the highest sensitivity through the whole sensing range and to build a model that simulates the behavior of the sensor under vacuum. A completely new design and a model simulating the behavior of the sensor from high vacuum to atmospheric pressure were established.

## 1. Introduction

Vacuum technology needs accurate pressure monitoring for different kinds of purposes, including thermal insulation and correct operation of manufacturing systems. Applications might vary from simply monitoring all or parts of a pump down cycle, carefully measuring and controlling a stringent target pressure, or supervising a critical industrial process. In the semiconductor industry, for instance, this enabled dramatic progress on several frontiers of contemporary electronics such as lifetime, precision, and reproducibility of devices that are currently ubiquitous. This capability originates essentially from better production and process control relying on, amongst others, accurate pressure sensors. However, the diversity of pressure ranges and of the required accuracies annihilates the possibility of implementing a single type of gauge that will be of use to all requirements. That is why several types of vacuum sensors based on different operating principles are commercially available operating from as low as 10^−10^ Pa up to atmospheric pressure and far above. Nonetheless, a cheap sensor that would extend into the high vacuum range and tolerate atmospheric or even higher pressure without switching is highly desirable and not commercially available. Table 1 shows the degree of vacuum with respect to pressure.

Conventional systems for measuring sub-atmospheric pressures include mechanical manometers, ionization gauges, and heat conductivity manometers [1]. Mechanical manometers use some solid deflectable objects [2] such as tubes, plates, or diaphragms to measure pressure. The system whose pressure is to be measured is connected to the deflecting object. Any change in pressure causes the object to deflect and this deflection is mechanically amplified using a suitable gear and linkage mechanism, indicated on a calibrated dial. Ionization gauges are vacuum gauges in which the pressure is indicated by the ionization current between two specified electrodes at a prescribed voltage [1].

A heat conductivity pressure gauge is based on a heating element (wire, plate, or chip) inserted inside a chamber that transfers thermal energy to any gas molecules that come into contact with it, and that energy is again transferred to the walls of the chamber. With continuous motion of the gas molecules, a thermal equilibrium is reached as long as the number of gas molecules, i.e., the pressure, remains constant.

If, however, the pressure changes while the wire is being heated from a constant power source, a new thermal equilibrium is reached, and the temperature of the wire changes to indicate the new number of gas molecules that can carry heat away from it.

This means that the temperature of the wire can be used as an indication of the pressure inside the chamber. This is the basic principle of all thermal conductivity gauges to which Pirani sensors belong. They are known for their unsurpassed accuracy in the rough vacuum area. In practice, the heated element is an electrical resistance inserted inside a Wheatstone bridge. Therefore, the temperature variation is transduced into a precise voltage variation.

## 2. Theoretical Background

The Pirani principle operates when a heated element is inserted inside a vacuum chamber. When the Pirani element is supplied with a constant heating power P, it will transfer heat to its surroundings and reach an equilibrium temperature characteristic of the pressure. The Pirani element transfers heat by three means:

- Solid conduction from the Pirani sensor to its carrier; the value of the thermal conductance depends on the geometry of the sensor’s carrier and its thermal conductivity.
- Radiation from the sensor’s hot surface to the surface of the chamber; its value depends on the emissivity and the exterior surface of the sensor.
- Solid to gas conduction from the sensor to the gas molecules that contact it, which effectively depends on pressure.

The equilibrium equation can be expressed as [3]

(1) $$P=\left(T-T_{0}\right)\left(G_{r}+G_{c}+G_{g}\left(p\right)\right)$$

where P is the heating power supplied to the sensor, T is the equilibrium temperature, $T_{0}$ is the initial temperature, $G_{r}$ is the radiative thermal conductance, $G_{c}$ is the solid thermal conductance of the sensor’s carrier, and $G_{g}$ is the gas conductance.

(2) $$G_{r}=\frac{\varepsilon \sigma \left(T^{4}-T_{0}^{4}\right)A}{\left(T-T_{0}\right)}$$

where ε is the emissivity of the sensor exterior surface, σ is the Stefan–Boltzmann constant, and A is the sensor’s emitting surface.

In the case where the sensor is suspended by one cylindrical wire, the solid conductance can be expressed as [4]

(3) $$G_{c}=\frac{\lambda \pi \cdot r^{2}}{l}$$

where λ is the thermal conductivity of the wire material, r is the radius of the wire, and l is the length of the wire. The gas conductance is expressed as follows [3]:

(4) $$G_{g}\left(p\right)=\lambda \left(p,h\right)\frac{A}{h},$$

with λ(p,h) the thermal conductivity of the gas at pressure p, and A the sensor’s surface.

(5) $$\lambda \left(p,h\right)=\lambda \left(p_{0}\right){\left(1+2\cdot \frac{\left(2-a\right)\bar{l}\left(p\right)}{a\cdot h}\frac{9.5}{6}\right)}^{-1},$$

where $\lambda (p_{0})$ represents the thermal conductivity of the gas at atmospheric pressure, a is the energy accommodation coefficient of the gas molecules, $\bar{l}(p)$ is the pressure-dependent mean free path of the gas molecules, and h is the distance between the sensor’s heated surface and the cold ambient surface.

**Figure 1:**
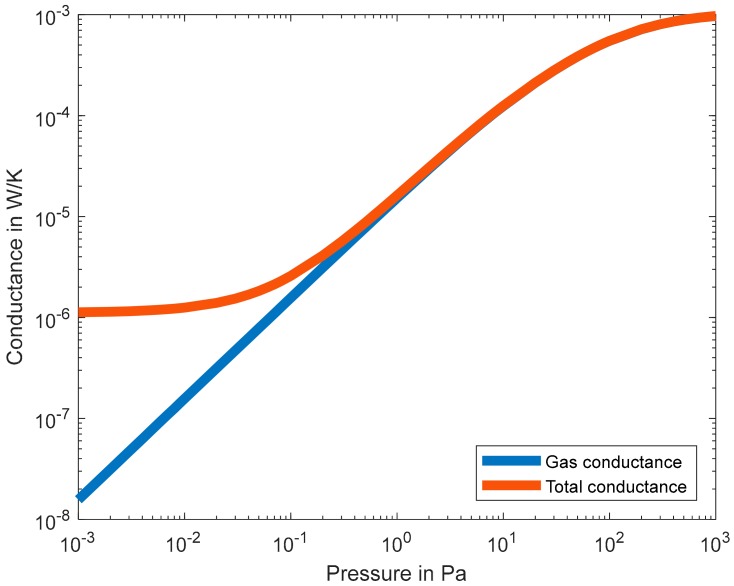
Ratio of gas conductance to total conductance versus pressure computed from Reference [3].

**Figure 2:**
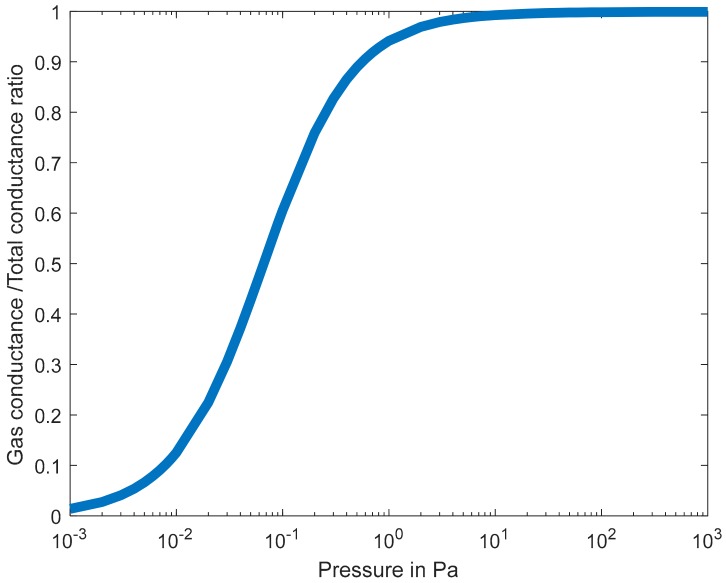
Values of gas conductance and total thermal conductance vs. pressure computed from Reference [3].

The change in pressure vs. wire temperature remains fairly linear over a pressure range of about 0.01 Pa to 100 Pa depending on the wire dimensions. Below this range, heat transfer is dominated by radiation from the wire’s surface and conduction from the wire to its carrier [3,5,6]. Above this range, heat transfer is ruled by thermal convection [7]. In addition to that, other heat transfer mechanisms, i.e., solid conduction from chip to carrier and radiation, overwhelm the pressure-dependent gas heat transfer. Figure 1 shows the ratio of gas conductance over total conductance versus pressure (obtained from Reference [3]). It can be seen that, below 10^−2^ Pa, the gaseous conductance represents less than 10% of the total conductance and, above 100 Pa, it represents more than 99% of the total conductance. Figure 1 shows the actual values of the gas conductance and the total conductance computed from Reference [3]. Figure 2 depicts the gaseous heat transfer over the total heat transfer for the device introduced in Reference [3].

A wide choice of sensors based on the Pirani principle is available in the literature. A list of references is given in Table 2, which also includes the measurement principles and the range of the sensors. The devices are often made using microfabrication and semiconductor technologies [8]. These sensors address different pressure ranges: from two decades of pressure up to seven decades of pressure for one single device [5]. 

However, systematic investigations of Pirani wires identified limits of the process corresponding to saturation due to the pressure dependence of thermal conductivity in high vacuum and close to atmospheric pressure [5,8].

A high vacuum process will need to be provided with gauging that follows the pump down cycle from atmospheric pressure through the volume zone and into the dry down zone. A thermal conductivity gauge can follow the pressure all the way through the volume zone; however, when the system goes into the dry down zone below about 10^−2^ Pa, where water vapor becomes the predominant residual gas, an ionization gauge becomes necessary. Usually, with the exception of some extended range gauge modifications, these two gauges together can be used to cover the full pump down cycle. This is why many electronic gauge controllers combine both types of gauges in the same unit. For instance, vacuum hybrid sensors such as Pirani Bayard–Alpert and Pirani–Magnetron increase the measurement range from atmospheric to the ultra-high vacuum. Consequently, the revived development of hybrid sensors combining two or more operating principles received much attention recently, since many combined Pirani gauges are commercialized (Canon Cold Cathode Pirani Gauge M-360 CP or BCG 450 from INFICON, for instance). In the present case, two different measurement techniques will be combined, namely the miniaturized Pirani principle and Surface Acoustic Waves (SAW).

**Figure 3:**
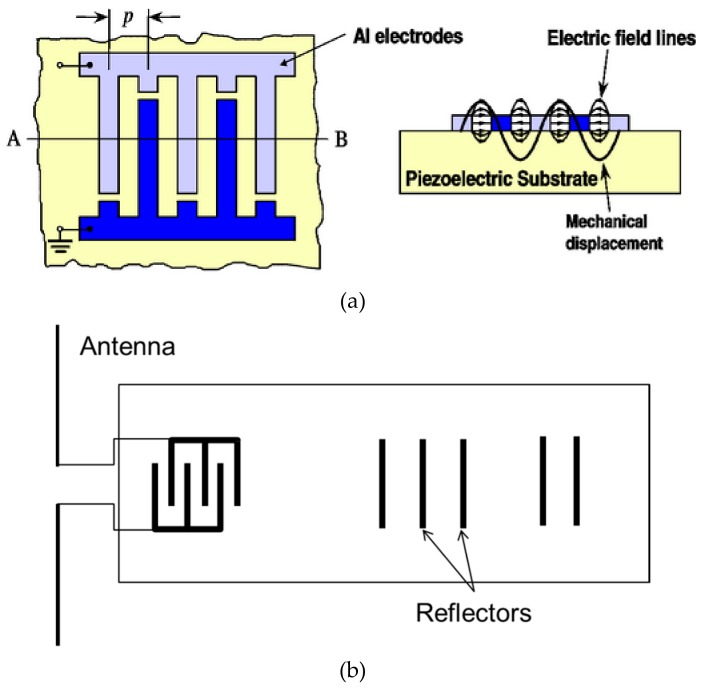
(**a**) Schematic structure of a surface acoustic wave (SAW) device. (**b**) A SAW interdigitated transducer (IDT) with its reflector and antenna. Source Carinthian Tech Research.

Surface acoustic waves are elastic waves that propagate on the surface of piezoelectric crystals [2]. Their propagation frequency and elastic constants are sensitive to the surrounding environment´s properties including temperature, pressure, and humidity. SAW devices and sensors are made of a piezoelectric crystal substrate with an interdigitated transducer (IDT) on its surface that converts voltage or other signals to SAW back and forth. An interdigitated transducer consists of a series of etched metallic electrodes whose dimensions determine the properties of the waves that they will generate. SAW devices offer opportunities for wireless operation, as well as self-heating effects [33,34]. Having an interdigitated transducer printed on their surface enables them to send and receive waves. Figure 3 shows the principle structure of a SAW device. The SAW IDT is connected to an antenna, generating surface waves when a radio-frequency pulse signal is received. A series of reflectors is available. Reflected waves are received by the transducer, which makes the antenna radiate a return signal with characteristics determined by the reflectors. The reflector configuration can also be used for identifying the device. Many Industrial, Scientific and Medical radio bands are used to operate SAW devices, among which the 2.45-GHz used for the presented device. SAW devices can be operated passively with no need of power supply. Wireless operation is possible because the device can give relatively long delays, preventing overlap between interrogation signal and return signal.

Thanks to their sensitivity to pressure, SAW devices can also be used as pressure sensors. Pressure-induced bending of the piezoelectric crystal membrane generates a change in wave speed that is directly correlated to the pressure. This effect has good sensitivity over a restricted pressure range of one to two decades. Indeed, beyond two decades of pressure, the bending of the crystal will be too large and causes destruction of the crystal [35].

## 3. Sensor Description

Nicolay et al. first had the idea to combine both the Pirani effect and SAW to design a pressure sensor with extended range and sensitivity [33]. After promising first results, wireless heating was added [36]. The device was relatively bulky (10 mm × 3.1 mm × 8 mm only for the sensing chip) with a very high response time (more than 5 min). In the present work, a similar approach was adopted combining the Pirani principle and SAW.

### 3.1. Structure of the Sensor

**Figure 4:**
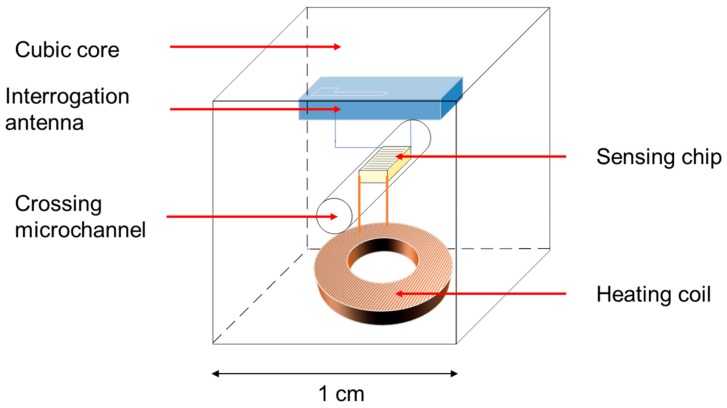
Structure of the wireless vacuum sensor. The design shows a 1 cm^3^ polymethylmethacrylate (PMMA) cube crossed at its center by a microchannel. The sensing SAW–Pirani chip is inserted inside the microchannel. The heating coil and the interrogation antenna are buried inside the core of the sensor.

The objective of the design work was to develop a sensor able to handle pressures between 10^−4^ Pa and atmospheric pressure, operating wirelessly and being suitable for microfluidic applications, as well as macroscopic industrial applications. Technical solutions were investigated to design a completely autonomous system fulfilling those requirements. The investigations resulted in the following structure: a 1-cm^3^ polymer cube crossed at its center by a microchannel used for pressure measurement. The heating coil is buried in the bottom part of the core of the sensor, and the interrogation antenna is screen-printed inside the core. Figure 4 shows a schematic of the device.

### 3.2. Components of the Sensor

The components of the sensor are as follows:

- A polymer housing crossed by the microchannel;
- A SAW–Pirani chip consisting of a block of lithium niobate with an interdigital transducer printed at its surface and a Joule resistance layer at its bottom;
- A heating coil encapsulated by liquid polymer which acts as a seal and controls the temperature;
- An interrogation antenna made with silver screen printing.

**Figure 5:**
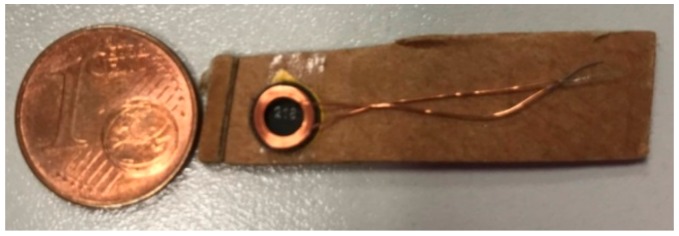
WE-WPCC 760308101216 wireless power charging receiver coil manufactured by Wuerth Elektronik.

A commercial coil was selected that fulfills the requirements of the sensor in terms of power transfer, size, and coupling distance. The chosen coil was the WE-WPCC 760308101216 wireless power charging receiver coil manufactured by Wuerth Elektronik (see Figure 5). It can be powered from a distance of up to 2 cm and delivers power of up to 0.11 W.

**Figure 6:**
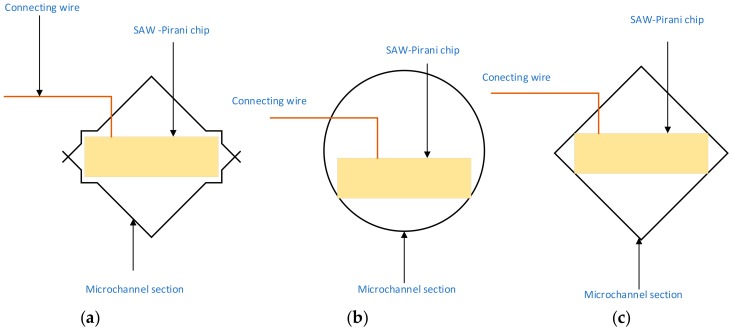
Different possible cross-sections of the microchannel crossing the sensor: (**a**) quadratic with holding cavities; (**b**) circular; (**c**) quadratic and size-matching.

The polymer housing is made of polymethylmethacrylate (PMMA). The polymer cube is crossed by a microchannel whose cross-section still needs to be determined. Figure 6 shows potential cross-sections of the channel. The cross-section should be designed on one hand in a way to minimize the physical contact between the chip and the channel, and, on the other hand, to mechanically stabilize and protect the chip. Indeed, the physical contact between the chip and the channel will generate heat leakages that could decrease the sensitivity of the sensor at high vacuum. Thus, the part of the microchannel in physical contact with the cube should be made from an insulating material such as the Code 9658 ceramic.

**Figure 7:**
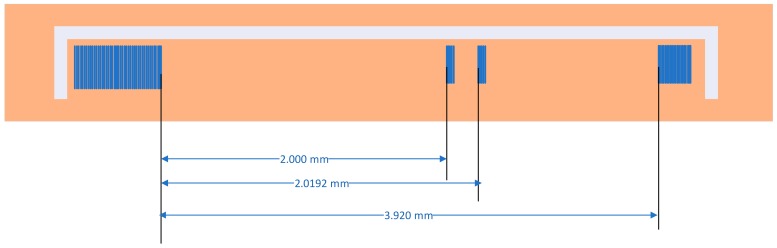
Geometry of the interdigitated transducer generated on top of the chip.

The dimensions of the chip block were set by Finite Element Method (FEM) heat transfer simulations, as well as the energy required to heat the chip. However, the advocated interdigitated transducer (IDT) on top of the chip was a new design that had to be generated with electron beam lithography. A Joule resistance was added to the chip where the coil delivers its power. Figure 7 shows the design of the IDT. Here, the resistance is surrounding the electrodes, but it can also be manufactured at the bottom of the chip. The structure of the IDT is similar to the one presented in Reference [37], where a SAW chip was used as a temperature sensor operating at 2.45 GHz. The corresponding wavelength is 1.3836 µm, the length of the electrodes is 30 wavelengths, i.e., 41.508 µm, and the main IDT contains 22 finger pairs with a metallization ratio of 50%. The thickness of the electrodes is around 50 nm. The first reflector contains five electrodes and is located 2.000 mm from the main IDT, the second reflector also contains five electrodes and is located 2.0192 mm from the main IDT, and the third reflector contains 20 electrodes and is located 3.920 mm from the main IDT.

### 3.3. Operation of the Sensor

**Figure 8:**
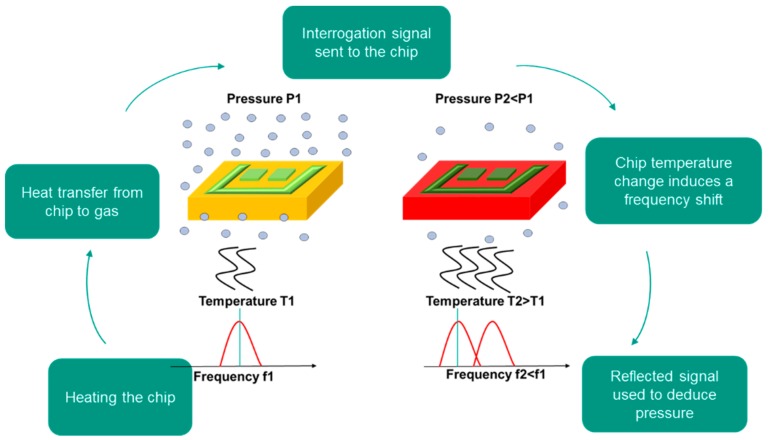
Operating protocol of the sensor.

The sensor is first inserted in a vacuum environment. Then, the SAW chip inside the microchannel is heated via the Joule resistance, receiving energy from the coil. The optimal heating mode of the chip, either pulsed or continuous, still needs to be determined. The interrogation signal is sent to the sensor via the interrogation antenna using a network analyzer. The reflected signal with the modified propagation frequency is received by the same interrogation antenna. The calibration of the sensor allows deducing the pressure from the frequency shift. Figure 8 shows a schematic of the operating protocol of the sensor.

### 3.4. Sensor Simulation

**Figure 9:**
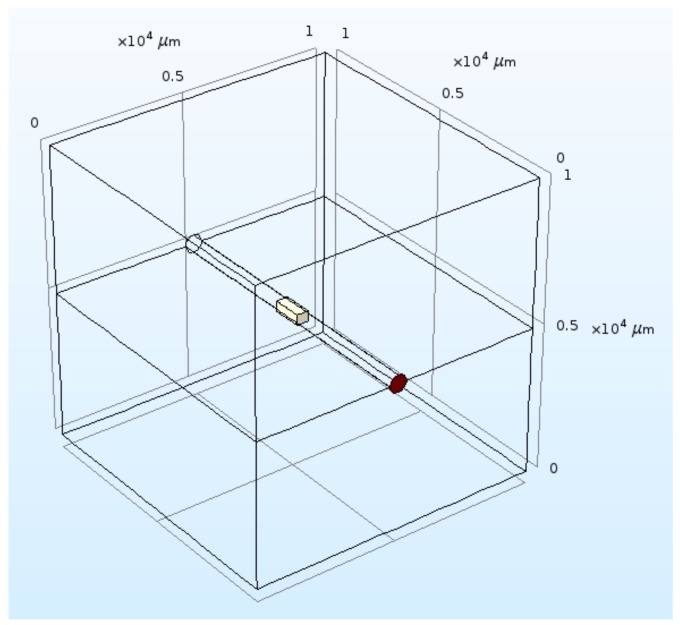
Geometry simulated in COMSOL.

The sensor designed was simulated using the software COMSOL. The thermal behavior in vacuum and the thermal response were simulated. Using the temperature response of the sensing chip, the corresponding frequency shift was calculated. The calibration curve of the sensor was computed. Figure 9 shows the geometry simulated: a 1-cm^3^ cube crossed at its center by a 600-µm-diameter microchannel. The chip was inserted inside the microchannel and had a length of 1 mm, a width of 400 µm, and a thickness of 350 µm. The pressure was simulated by the variation of the thermal conductivity of the gas. The values used are reported in Table 3.

**Figure 10:**
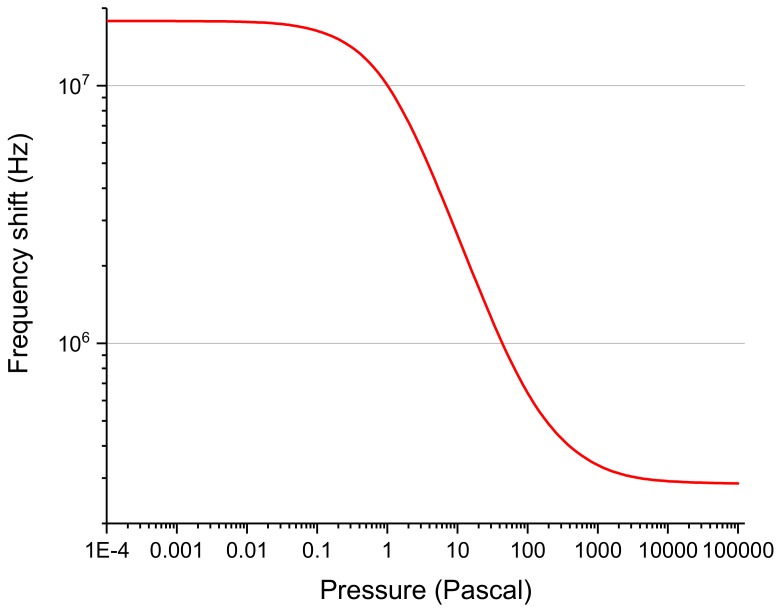
Calibration curve obtained from the simulation.

The thermal simulation enabled computing the calibration curve. Figure 10 plots the frequency shift corresponding to each pressure for an initial atmospheric pressure. Table 3 shows the frequency shift at different pressure ranges. The chip is constantly heated from its bottom by introducing a boundary heat source condition of 10,000 W/m^2^, which corresponds to a total power of 4 mW, and the steady-state temperature was observed with respect to pressure.

## 4. Discussion

### 4.1. Thermal Analysis

A thermal analysis using gas kinetic theory was performed for the present work. Since the sensor measures the pressure of a gas, it was inserted inside a gaseous environment with potential gas flows. To characterize this flow, some figures of merit were used to determine the dimensions of the device. The first figure of merit used was the Knudsen number Kn.

(6) $$Kn=\frac{\bar{l}(p)}{d},$$

where $\bar{l}(p)$ is the mean free path of the gas molecules in the conditions of the flow (temperature and pressure), and *d* is a characteristic dimension of the microchannel, here taken as the hydraulic diameter. The study gas here was nitrogen. The flow regimes were distinguished according to the value of the Knudsen number [38].

- For Kn<0.001, a continuum flow is taking place and is accurately modeled by the compressible Navier–Stokes equations with classical no-slip boundary conditions.
- For 0.001<Kn<0.1, the flow is a slip flow and the Navier–Stokes equations remain applicable, provided a velocity slip and a temperature jump are taken into account at the walls. Rarefaction effects become sensitive at the walls first.
- For 0.1<Kn<10, the flow is a transition flow. The intermolecular collisions are not yet negligible and have to be taken into account.
- For Kn>10, the flow is a free molecular flow and the occurrence of intermolecular collisions is negligible compared to the collisions of the gas molecules with the walls.

The second figure of merit needed for this analysis is the thermal conductivity, effectively depending on pressure. As stated in Völklein et al. [3], the thermal conductivity λ(p,h) of the gas depends upon the pressure-dependent mean free path of the gas molecules and the distance h between the hot chip and cold channel surface. This model was chosen for its simplicity and reliability, considering the good agreement obtained between simulation and experiments in Reference [23]. The whole sensing process was based on the thermal conductivity of the gas, as given in Equation (7).

(7) $$\lambda \left(p,h\right)=\lambda \left(p_{0}\right){\left(1+2\left(\frac{2-a}{a}\right)\frac{\bar{l}(p)}{h}\frac{9.5}{6}\right)}^{-1},\;$$

where $\lambda \left(p_{0}\right)$ represents the thermal conductivity of the gas at atmospheric pressure $p_{0}$, and a is the energy accommodation coefficient of the gas molecules at both surfaces (a=0.77 for $N_{2}$ molecules at smooth surfaces). For low pressures in the case of molecular flow, the thermal conductivity $\lambda \left(p,h\right)$ becomes directly proportional to ph, which is the pressure in Torr multiplied by the characteristic dimension d (see Equation (6)) [3].

(8) $$\lambda (p,h)=92\frac{W}{mK}\cdot \frac{ph}{mTorr}.$$

In the considered case, the Knudsen number is the ratio between the mean free path of the gas and the characteristic dimension of the system. The mean free path was calculated at a temperature of 300 K, while the pressure was varied between 10^−4^ Pa and 10^5^ Pa. The Pirani effect is based on the pressure dependence of the thermal conductivity of a gas at the transition and slip flow. The characteristic S-shaped curve of the thermal conductivity vs. pressure shows high sensitivity for pressures between 10^−2^ and 10^2^ Pa, with slight variations depending on the dimensions of the sensing element, as can be seen in Figure 9.

At the edges of this curve, i.e., at high vacuum and near atmospheric pressure, the behavior asymptotically corresponds to saturation. At high vacuum, the collisions between gas molecules and walls are too rare, and the heat transfer between the hot surface and the gas molecule is very small; it cannot be detected by measuring devices. Near atmospheric pressure, the collisions between the walls and the gas molecules are too frequent. This affects the sensitivity of the thermal conductivity to pressure and makes the Pirani effect less efficient. Moreover, near high vacuum, radiative heat transfer between the solid surfaces of the Pirani sensor and solid conduction are ruling the gaseous heat transfer, which makes it more difficult to acquire signals directly linked to the gas pressure.

Both Knudsen number and thermal conductivity were calculated using the mean free path of the gas molecules. It is important to have in mind the orders of magnitude of the mean free path at the different pressure levels. Table 4 shows the values of the mean free path of nitrogen at 300 K at pressure between 10^-4^ Pa and 10^5^ Pa. The mean free path was calculated using a hard sphere model presented in Reference [39]. It ranges from 11 m down to 0.6 nm. In order to achieve a high sensitivity of the Pirani effect, the Knudsen number should be between 0.001 and 10. With a single device, it is not possible to change the size of the gas gap with the pressure; thus, it is important to find a compromise with a dimension that will grant reasonable sensitivity through the whole pressure range.

**Figure 11:**
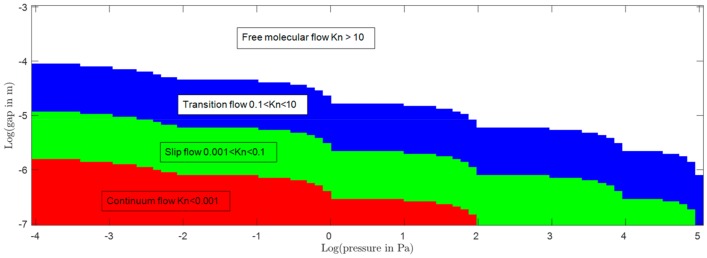
This figure shows the flow regime with respect to the pressure and the size of the gas gap where the sensor chip is located. The red area corresponds to the free molecular flow. The white area corresponds to the transition flow, the green area corresponds to the slip flow, and the blue area corresponds to the continuum flow.

Figure 11 shows the Knudsen regime with respect to the characteristic length of the channel and pressure for nitrogen. As already mentioned, the Pirani effect operates best at the transition and slip flow (green and white zones in the Figure 11), which gives an estimation of the optimum dimensions of the gas gap that need to be used to sense the complete range between high vacuum and atmospheric pressure. A 600 µm diameter was chosen to be the characteristic dimension of the microchannel described here for the first simulation so as to increase the sensitivity in high vacuum and for manufacturability concerns.

The structural geometry of the new sensor was simulated using the software COMSOL Multiphysics. A chip with dimensions of 6 mm length by 400 µm width by 350 µm thickness was inserted inside a microchannel with a diameter of 600 µm. The corresponding mean free path and thermal conductivities at 300 K were computed using Equations (7) and (8) stated above, which led to the values listed in Table 5. For comparison, a second chip was simulated providing 6 mm length by 400 µm width by 200 µm thickness. The thermal response of the sensor differed depending on the pressure. Indeed, for the same heating power of the device, the steady-state temperature and the transient response depend on the value of the pressure. This was the case at high vacuum, as well as near atmospheric pressure.

The values given in Table 5 are indicative, since the accommodation coefficients used to compute the thermal conductivity are not known precisely for this surface. However, the real values are expected to have the same order of magnitude, which grants this simulation qualitative relevance concerning the behavior of a real SAW–Pirani chip.

The thermal conductivity analysis and the simulation helped design the complete structure of the sensor shown in Figure 4. Using simulation results, the energy required to heat the chip was predicted to be approximately 24 mW. The simulation allowed determining the dimensions of the chip that provide acceptable sensitivity in high vacuum and atmospheric pressure, i.e., a temperature variation between pressures that can be easily measured wirelessly with the existing equipment, higher or equal to 0.1 K. Packaging and design solutions were chosen based on portability, size, and cost concerns.

### 4.2. Wireless Transmission Methods

Operating a Pirani sensor in a completely wireless way provides major advantages compared to conventional techniques. It is no longer necessary to contact any heating element with wires or to read-out by physical contacts, which avoids leakage in the vacuum range. This makes vacuum-tight throughput systems obsolete for this sensor. Nevertheless, designing the heating element, as well as the read-out element, in a wireless way gives a real novel approach to this sensor type. With this in mind, Surface Acoustic Waves were selected, considering the fact that it is possible to interrogate SAW devices wirelessly for a long time and the technologies are well known. However, heating micro devices wirelessly is still not widespread. Some efforts were taken to find a wireless heating solution for the sensor complying with the size restrictions. Table 6 lists different kinds of wireless power supplies already commercially available.

### 4.3. Choice of the Interrogation Frequency and Interrogation Antenna

While wireless interrogation can be achieved at any readout frequency, there is only a distinct number of radio-frequency (RF) bands which are free for industrial–scientific medical (ISM) applications. Here, the ISM band from 2.4 GHz to 2.4835 GHz proved to be most suitable, as it has an adequate bandwidth (83.5 MHz) and an almost worldwide geographical license. At the same time, it allows a read-out at a distance of several meters. At this frequency, the radio-frequency wavelength is about 13.5 cm, thus permitting the use of simple and small antennas, e.g., dipoles, and slot or patch antennas, favorably for the transponder part. To achieve higher precision, the frequency of 2.45 GHz was chosen to allow a high frequency shift which is more easily detectable especially at low pressures. The stated precision of lithium niobate SAW devices is 70 to 90 ppm/K [40] and up to 100 ppm/K, which represents 30,310 to 38,970 Hz/K at 433 MHz and 171,500 to 220,500 Hz/K at 2.45 GHz. After the choice of the interrogation frequency, the corresponding antenna has to be designed and inserted inside the cube. COMSOL will be used to optimize the shape, size, and position of the antenna inside the cube.

## 5. Conclusions

The vacuum sensor presented here is new in the sense that it is extending the sensing range, while operating completely wirelessly. The performance was not tested in real-world applications, but it contains innovations compared to conventional Pirani wires or SAW sensors or even the SAW–Pirani sensors presented in the literature. The prototype still needs to be assembled and tested, which will state the actual performance of the sensor. Previous work on SAW–Pirani sensors was studied, and design directions such as the reduction of the thickness of the chip, the increase of the surface-to-volume ratio, and the increase of the operating frequency to obtain a higher thermal sensitivity of the chip were taken into account.

This work presents an innovative compact wireless SAW–Pirani sensor for microscopic and macroscopic applications. The combination of both surface acoustic waves and heat transfer in gases allows extending the sensing range of a vacuum sensor and to operate this sensor wirelessly, which can be advantageous in vacuum technology. The results given by simulation predict a sensitivity of 0.1 K for a pressure variation of 10^−4^ Pa, which could easily be detected by the available instruments in the lab. Furthermore, it is expected that further investigations concerning the modus operandi of the sensor will improve the sensitivity of the sensor in high vacuum and close to atmospheric pressure. For instance, applying pulsed heating instead of continuous heating could increase the sensitivity of the resonance frequency of the sensor to the pressure through the whole target range. A prototype of the described sensor design is currently being assembled and tested. The best modus operandi of the sensor still needs to be determined. 

Further work, therefore, includes the investigation of the transformation of SAW signal in vacuum and its dependence on pressure. To support that, Sharipov et al. reported interaction between acoustic waves and thermal waves in vacuum [41]. 

## Figures and Tables

**Table 1:** Degree of vacuum with respect to pressure.

Pressure Range in Pa	Degree of Vacuum
10^5^ to 3 × 10^3^	Low vacuum
3 × 10^3^ to 10^−1^	Medium vacuum
10^−1^ to 10^−4^	High vacuum
10^−4^ to 10^−7^	Very high vacuum
10^−7^ to 10^−10^	Ultra-high vacuum (UHV)
<10^−10^	Extreme-ultrahigh vacuum (EHV or XHV)

**Table 2:** Detection principles and pressure ranges of micro-electro-mechanical system (MEMS) Pirani gauges.

Researcher	Type of Gauge	Pressure Range (Pa)
Van Herwaarden and Sarro, 1988 [2]	Heated cantilever combined with thermopile	0.13–13300
Völklein and Schnelle, 1991 [7]	Heated resistor combined with thermopile	0.13–10
Piotto et al., 2017 [9]	Heated resistor with thermopile	0.3–10^5^
Mastrangelo and Muller, 1991 [8]	Microbridge	10–10000
Swart et al., 1994	Microbridge	13–1.33 × 10^5^
Chae et al., 2004	Microbridge	2.6–267
Moelders et al., 2004	Microbridge	1.33–133
Doms et al., 2005 [10]	Microbridge	100–10^5^
Stark et al., 2005	Microbridge	1.33–10^6^
Mitchell et al., 2008 [11]	Microbridge	1.33–10^5^
Khosraviani and Leung, 2009 [12]	Microbridge	13.3–10^6^
Li et al., 2010 [13]	Microbridge	106–26,665
Jiang et al., 2010 [14]	Microbridge	0.1–1000
Chen, 2012 [15]	Microbridge	133–1.33 × 10^5^
Puers et al., 2002 [16]	Microbridge	100–10^7^
Moutaouekkil et al., 2015 [17]	Microbridge	1000–10^5^
Mailly et al., 2009 [18]	Microbridge	20–20,000
Robinson et al., 1992 [8]	Resistor on dielectric membrane	10–13,300
Paul et al., 1994 [19]	Resistor on dielectric membrane	100–10^5^
Shie et al., 1995 [20]	Resistor on dielectric membrane	1.33 × 10^−5^–133
Chuo et al., 1997 [21]	Resistor on dielectric membrane	13.3–1.33 × 10^7^
Stark et al., 2003	Resistor on dielectric membrane	1.33–13,300
De Jong et al., 2003	Resistor on dielectric membrane	10–20,000
Zhang et al., 2006 [22]	Resistor on dielectric membrane	10–10^5^
Völklein et al., 2013 [23]	Resistor on dielectric membrane	1.33 × 10^−4^–1332
Grau et al., 2014 [6]	Resistor on dielectric membrane	0.13–10^5^
Xiao	Resistor on dielectric membrane	1–1000
Kimura et al., 2007 [2]	Resistor on dielectric membrane	0.002–10^5^
Jeon et al., 2016 [24]	Resistor on dielectric membrane	0.013–10^5^
Paul and Baltes, 1995 [25]	Resistor on dielectric membrane	100–10^6^
Wenzel and Bak, 1998 [26]	Resistor on diaphragm	10–10^5^
Qui et al., 2009	Metallic wire	1–100
Brun et al., 2012 [27]	Silicon nanowire	50–10^5^
Ghouila-Houri et al., 2017 [28]	Microwire	10,000–8 × 10^5^
Schelcher et al., 2011 [29]	Ni-microbeam	3.3–10^5^
Wang et al., 2011 [30]	Microplate	0.1–10^5^
Santagata et al., 2011 [31]	Tube-shaped	0.133–1.33 × 10^5^
Mercier et al., 2012 [32]	Cr/Au-resistor on LiNbO_3_-substrate (SAW device)	0.001–10^5^

**Table 3:** Frequency shift of the sensor at different pressure ranges.

Pressure Range	Frequency Shift
10^−4^ to 10^−3^ Pa	15.435 kHz
10^−3^ to 10^−2^ Pa	152.145 kHz
10^3^ to 10^4^ Pa	55.125 kHz
10^4^ to 10^5^ Pa	6.615 kHz

**Table 4:** Mean free path of nitrogen at 300 K for pressures between 10^−4^ Pa and 10^5^ Pa.

Pressure	Mean Free Path	Minimum Size for Knudsen Number below 10
0.0001 Pa	11.4435 m	1.14 m
0.001 Pa	1.144 m	0.114 m
0.01 Pa	0.1144 m	0.01144 m
0.1 Pa	0.0114 m	0.00114 m
1 Pa	0.0011 m	0.00011 m
10 Pa	1.1444 × 10^−4^ m	1.1444 × 10^−5^ m
100 Pa	1.1444 × 10^−3^ m	1.1444 × 10^−4^ m
1000 Pa	1.178 µm	117.8 nm
10,000 Pa	117.8 nm	11.78 nm
50,000 Pa	23.6 nm	2.36 nm
100,000 Pa	11.8 nm	1.18 nm
101,325 Pa	11.6 nm	1.16 nm
200,000 Pa	5.9 nm	5.9 Å

**Table 5:** Thermal conductivity values used for the simulation.

Pressure	Thermal Conductivity (W/m/K)
**High Vacuum**	
0.0001 Pa	4.9 × 10^−7^
0.0002 Pa	9.8 × 10^−7^
0.0003 Pa	1.47 × 10^−6^
0.0005 Pa	2.45 × 10^−6^
0.001 Pa	4.90 × 10^−6^
**Near Atmospheric Pressure**	
1000 Pa	0.0163
10,000 Pa	0.0232
50,000 Pa	0.0245
100,000 Pa	0.0246
200,000 Pa	0.0252

**Table 6:** Wireless transfer methods available in the market.

Energy Coupling	Magnetic Induction	Magnetic Resonance	Electrostatic Coupling	Wireless Transmission
**Description**	Inductive coupling between 2 coils	Coupling between 2 tuned oscillating circuits	Capacitive coupling between 2 electrodes	Reception of radio waves and rectification
**Distance**	Few mm to 10 cm	Few cm to m	Few mm to few cm	Up to a few m
**Transferable power**	Few W to several kW	Few W to kW	Few W to few 100s of W	1 W max
**Electrical efficiency**	70% to 90% heat loss	40% to 60% residual heat	60% to 90% heat loss	
**Frequency**	10 kHz	Few 100 kHz up to MHz	Few 100 kHz to few MHz	Frequencies up to microwaves
