# Correction: Design and Simulation of a Wireless SAW–Pirani Sensor with Extended Range and Sensitivity

**DOI:** 10.3390/s19143243

**Published:** 2019-07-23

**Authors:** Sofia Toto, Pascal Nicolay, Gian Luca Morini, Michael Rapp, Jan G. Korvink, Juergen J. Brandner

**Affiliations:** 1 Institute of Microstructure Technology, Karlsruhe Institute of Technology, Hermann-von-Helmholtz-Platz 1, 76344 Eggenstein-Leopoldshafen, Germany; 2 Carinthia Institute for Smart Materials and Manufacturing Technologies (CiSMAT), Carinthia University of Applied Sciences, 9524 Villach/St. Magdalen, Austria; 3 Dipartimento di Ingegneria Industriale, Alma Mater Studiorum Università di Bologna, Viale Risorgimento 2, I-40136 Bologna, Italy

The authors wish to make the following erratum to Reference [1]:

The Table 2 below contained false reference numbers. The references were corrected. The corrected references are also available below.

The authors would like to apologize for any inconvenience caused to the readers by these changes.

**Corrected references for Reference [1]**

1. Figliola, R.S.; Beasley, D.E. Theory and design for mechanical measurements. *Meas. Sci. Technol.* **1996**, *12*, 1743.
2. Pace, E.L. Scientific foundations of vacuum technique (Dushman, Saul). *J. Chem. Educ.* **1962**, *39*, A606.
3. Völklein, F.; Grau, M.; Meier, A.; Hemer, G.; Breuer, L.; Woias, P. Optimized MEMS Pirani sensor with increased pressure measurement sensitivity in the fine and high vacuum regime. *J. Vac. Sci. Technol. A Vac. Surf. Film.* **2013**, *31*, 061604.
4. Jitschin, W.; Ludwig, S. Dynamical behaviour of the Pirani sensor. *Vacuum* **2004**, *75*, 169–176.
5. Weng, P.K.; Shie, J.S. Micro-Pirani vacuum gauge. *Rev. Sci. Instrum.* **1994**, *65*, 492–499.
6. Grau, M.; Völklein, F.; Meier, A.; Kunz, C.; Kaufmann, I.; Woias, P. Optimized MEMS Pirani sensor with increased pressure measurement sensitivity in the fine and rough vacuum regimes. *J. Vac. Sci. Technol. A Vac. Surf. Film.* **2014**, *33*, 021601.
7. Xiao, B.; Dong, T.; Halvorsen, E.; Yang, Z.; Zhang, Y.; Hoivik, N.; Gu, D.; Tran, N.M.; Jakobsen, H. Integrated micro Pirani gauge based hermetical package monitoring for uncooled VOxbolometer FPAs. *Microsyst. Technol.* **2011**, *17*, 115–125.
8. Völklein, F.; Meier, A. Microstructured vacuum gauges and their future perspectives. *Vacuum* **2007**, *82*, 420–430.
9. Van Herwaarden, A.W.; Sarro, P.M. Performance of integrated thermopile vacuum sensors. *J. Phys. E Sci. Instrum.* **1988**, *21*, 1162–1167.
10. Völklein, F.; Schnelle, W. A vacuum microsensor for the low-vacuum range. *Sens. Mater.* **1991**, *3*, 41–48.
11. Piotto, M.; Del Cesta, S.; Bruschi, P. A Compact CMOS Compatible micro-Pirani Vacuum Sensor with Wide Operating Range and Low Power Consumption. *Procedia Eng.* **2016**, *168*, 766–769.
12. Mastrangelo, C.H.; Muller, R.S. Microfabricated thermal absolute-pressure sensor with on-chip digital front-end processor. *IEEE J. Solid-State Circuits* **1991**, *26*, 1998–2007.
13. Swart, N.R.; Nathan, A. An integrated CMOS polysilicon coil-based micro-Pirani gauge with high heat transfer efficiency. In Proceedings of the 1994 IEEE International Electron Devices Meeting, San Francisco, CA, USA, 11–14 December 1994; pp. 135–138.
14. Chae, J.; Stark, B.H.; Najafi, K. A micromachined Pirani gauge with dual heat sinks. In Proceedings of the 17th IEEE International Conference on Micro Electro Mechanical Systems. Maastricht MEMS 2004 Technical Digest, Maastricht, The Netherlands, 25–29 January 2004; pp. 532–535.
15. Moelders, N.; Daly, J.T.; Greenwald, A.C.; Johnson, E.A.; McNeal, M.P.; Patel, R.; Pralle, M.U.; Puscasu, I. *Micro and Nanosystems Symposium, Boston 2004*; Society, M.R., Ed.; MRS: Boston, MA, USA, 2004; p. 211.
16. Doms, M.; Bekesch, A.; Mueller, J. A microfabricated Pirani pressure sensor operating near atmospheric pressure. *J. Micromech. Microeng.* **2005**, *15*, 1504–1510.
17. Stark, B.H.; Junseok, C.; Kuo, A.; Oliver, A.; Khalil, N. A high-performance surface-micromachined Pirani gauge in SUMMIT V/spl trade. In Proceedings of the 18th IEEE International Conference on Micro Electro Mechanical Systems, 2005. MEMS 2005, Miami Beach, FL, USA, 30 January–3 February 2005; pp. 295–298.
18. Mitchell, J.; Lahiji, G.R.; Najafi, K. An Improved Performance Poly-Si Pirani Vacuum Gauge Using Heat-Distributing Structural Supports. *J. Microelectromech. Syst.* **2008**, *17*, 93–102.
19. Khosraviani, K.; Leung, A.M. The nanogap Pirani—A pressure sensor with superior linearity in an atmospheric pressure range. *J. Micromech. Microeng.* **2009**, *19*, 045007.
20. Li, Q.; Goosen, J.F.L.; van Beek, J.T.M.; van Keulen, F. A novel SOI Pirani sensor with triple heat sinks. *Procedia Chem.* **2009**, *1*, 160–163.
21. Jiang, W.; Wang, X.; Zhang, J. A single crystal silicon micro-Pirani vacuum gauge with high aspect ratio structure. *Sens. Actuators A Phys.* **2010**, *163*, 159–163.
22. Chen, C. Characterization of Gas Conductance of a Thermal Device with a V-Groove Cavity. *IEEE Electron Device Lett.* **2012**, *33*, 275–277.
23. Puers, R.; Reyntjens, S.; De Bruyker, D. The NanoPirani—An extremely miniaturized pressure sensor fabricated by focused ion beam rapid prototyping. *Sens. Actuators A Phys.* **2002**, *97–98*, 208–214.
24. Moutaouekkil, M.; Talbi, A.; Viard, R.; Gerbedoen, J.C.; Okada, E.; Elmazria, O.; Preobrazhensky, V.; Merlen, A.; Pernod, P.; Joint International Laboratory LIA LICS/LEMAC. Elaboration of a Novel Design Pirani Pressure Sensor for High Dynamic Range Operation and Fast Response Time. *Procedia Eng.* **2015**, *120*, 225–228.
25. Mailly, F.; Dumas, N.; Pous, N.; Latorre, L.; Garel, O.; Martincic, E.; Verjus, F.; Pellet, C.; Dufour-Gergam, E.; Nouet, P. Pirani pressure sensor for smart wafer-level packaging. *Sens. Actuators A Phys.* **2009**, *156*, 201–207.
26. Robinson, A.M.; Haswell, P.; Lawson, R.P.W.; Parameswaran, M. A thermal conductivity microstructural pressure sensor fabricated in standard complementary metal-oxide semiconductor. *Rev. Sci. Instrum.* **1992**, *63*, 2026–2029.
27. Paul, O.; Haberli, A.; Malcovati, P.; Baltes, H. Novel integrated thermal pressure gauge and read-out circuit by CMOS IC technology. In Proceedings of the 1994 IEEE International Electron Devices Meeting, San Francisco, CA, USA, 11–14 December 1994; pp. 131–134.
28. Shie, J.S.; Chou, B.C.S.; Chen, Y.M. High performance Pirani vacuum gauge. *J. Vac. Sci. Technol. A* **1995**, *13*, 2972–2979.
29. Jong, B.R.D.; Bula, W.P.; Zalewski, D.; Baar, J.J.V.; Wiegerink, R.J. Pirani pressure sensor with distributed temperature measurement. In Proceedings of the SENSORS, 2003 IEEE, Toronto, ON, Canada, 22–24 October 2003; Volume 1, pp. 718–722.
30. Zhang, F.T.; Tang, Z.; Yu, J.; Jin, R.C. A micro-Pirani vacuum gauge based on micro-hotplate technology. *Sens. Actuators A Phys.* **2006**, *126*, 300–305.
31. Takashima, N.; Kimura, M. Investigation on the Thin Film Pirani Vacuum Sensor Using A Constant Voltage Drive-Mode Diode-Heater. *IEEJ Trans. Sens. Micromach.* **2008**, *128*, 209–213.
32. Jeon, G.-J.; Kim, W.Y.; Shim, H.B.; Lee, H.C. Nanoporous Pirani sensor based on anodic aluminum oxide. *Appl. Phys. Lett.* **2016**, *109*, 123505.
33. Paul, O.; Baltes, H. Novel fully CMOS-compatible vacuum sensor. *Sens. Actuators A Phys.* **1995**, *46*, 143–146.
34. Wenzel, O.; Bak, C.K. The Micro Pirani™: A solid-state vacuum gauge with wide range. *Vak. Forsch. Prax.* **1998**, *10*, 298–301.
35. Qiu, Y.; Zhao, L.; Jin, Y. A novel micro pirani gauge with mono-wire sensing unit for microsystem application. In Proceedings of the 2009 International Conference on Electronic Packaging Technology & High Density Packaging, Beijing, China, 10–13 August 2009; pp. 467–470.
36. Brun, T.; Mercier, D.; Koumela, A.; Marcoux, C.; Duraffourg, L. Silicon nanowire based Pirani sensor for vacuum measurements. *Appl. Phys. Lett.* **2012**, *101*, 183506.
37. Ghouila-Houri, C.; Sindjui, R.; Moutaouekkil, M.; Elmazria, O.; Gallas, Q.; Garnier, E.; Merlen, A.; Viard, R.; Talbi, A.; Pernod, P. Nanogap Pirani Sensor Operating in Constant Temperature Mode for Near Atmospheric Pressure Measurements. *Proceedings* **2017**, *1*, 377.
38. Schelcher, G.; Fabbri, F.; Lefeuvre, E.; Brault, S.; Coste, P.; Dufour-Gergam, E.; Parrain, F. Modeling and characterization of MicroPirani vacuum gauges manufactured by a low-temperature film transfer process. *J. Microelectromech. Syst.* **2011**, *20*, 1184–1191.
39. Wang, X.; Liu, C.; Zhang, Z.; Liu, S.; Luo, X. A micro-machined Pirani gauge for vacuum measurement of ultra-small sized vacuum packaging. *Sens. Actuators A Phys.* **2010**, *161*, 108–113.
40. Santagata, F.; Iervolino, E.; Mele, L.; van Herwaarden, A.W.; Creemer, J.F.; Sarro, P.M. An analytical model and verification for MEMS Pirani gauges. *J. Micromech. Microeng.* **2011**, *21*, 115007.
41. Mercier, D.; Bordel, G.; Brunet-Manquat, P.; Verrun, S.; Elmazria, O.; Sarry, F.; Belgacem, B.; Bounoua, J. Characterization of a SAW-Pirani vacuum sensor for two different operating modes. *Sens. Actuators A Phys.* **2012**, *188*, 41–47.
42. Rokhlin, S.I.; Kornblit, L.; Gorodetsky, G. Surface acoustic wave pressure transducers and accelerometers. *Prog. Aerosp. Sci.* **1984**, *21*, 1–31.
43. Singh, K.J.; Elmazria, O.; Sarry, F.; Nicolay, P.; Ghoumid, K.; Belgacem, B.; Mercier, D.; Bounouar, J. Enhanced Sensitivity of SAW-Based Pirani Vacuum Pressure Sensor. *IEEE Sens. J.* **2011**, *11*, 1458–1464.
44. Joshi, S.G. Use of a surface-acoustic-wave (SAW) device to measure gas flow. *IEEE Trans. Instrum. Meas.* **1989**, *38*, 824–826.
45. Nicolay, P. Surface Acoustic Wave sensors: Applications for the Measurement of Low Pressures and High Temperatures. Ph.D. Thesis, Université Henri Poincaré, Nancy, France, 2007.
46. Nicolay, P.; Lenzhofer, M. A wireless and passive low-pressure sensor. *Sensors* **2014**, *14*, 3065–3076.
47. Fu, C.; Ke, Y.; Li, M.; Luo, J.; Li, H.; Liang, G.; Fan, P. Design and Implementation of 2.45 GHz Passive SAW Temperature Sensors with BPSK Coded RFID Configuration. *Sensors* **2017**, *17*, 1849.
48. Kandlikar, S.; Garimella, S.; Li, D.; Colin, S.; King, M.R. *Heat Transfer and Fluid Flow in Minichannels and Microchannels*; Elsevier Science: Oxford, UK, 2006.
49. Royer, D.; Dieulesaint, E. *Ondes Élastiques Dans les Solides*; Masson: Paris, France, 1996; p. 321.
50. Kalempa, D.; Sharipov, F. Numerical modelling of thermoacoustic waves in a rarefied gas confined between coaxial cylinders. *Vacuum* **2014**, *109*, 326–332.

## Figures and Tables

**Table 2:** Detection principles and pressure ranges of micro-electro-mechanical system (MEMS) Pirani gauges.

Researcher	Type of Gauge	Pressure Range (Pa)
Van Herwaarden and Sarro, 1988 [9]	Heated cantilever combined with thermopile	0.13–13,300
Völklein and Schnelle, 1991 [10]	Heated resistor combined with thermopile	0.13–10
Piotto et al., 2016 [11]	Heated resistor with thermopile	0.3–10^5^
Mastrangelo and Muller, 1991 [12]	Microbridge	10–10,000
Swart et al., 1994 [13]	Microbridge	13–1.33 × 10^5^
Chae et al., 2004 [14]	Microbridge	2.6–267
Moelders et al., 2004 [15]	Microbridge	1.33–133
Doms et al., 2005 [16]	Microbridge	100–10^5^
Stark et al., 2005 [17]	Microbridge	1.33–10^6^
Mitchell et al., 2008 [18]	Microbridge	1.33–10^5^
Khosraviani and Leung, 2009 [19]	Microbridge	13.3–10^6^
Li et al., 2009 [20]	Microbridge	10.6–26,665
Jiang et al., 2010 [21]	Microbridge	0.1–1,000
Chen, 2012 [22]	Microbridge	133–1.33 × 10^5^
Puers et al., 2002 [23]	Microbridge	100–10^7^
Moutaouekkil et al., 2015 [24]	Microbridge	1,000–10^5^
Mailly et al., 2009 [25]	Microbridge	20–20,000
Robinson et al., 1992 [26]	Resistor on dielectric membrane	10–13,300
Paul et al., 1994 [27]	Resistor on dielectric membrane	100–10^5^
Weng and Shie, 1994 [5]	Resistor on dielectric membrane	1.33 × 10^−5^–133
Shie et al., 1995 [28]	Resistor on dielectric membrane	13.3–1.33 × 10^7^
De Jong et al., 2003 [29]	Resistor on dielectric membrane	10–20,000
Zhang et al., 2006 [30]	Resistor on dielectric membrane	10–10^5^
Völklein et al., 2013 [3]	Resistor on dielectric membrane	1.33 × 10^−4^–1332
Grau et al., 2014 [6]	Resistor on dielectric membrane	0.13–10^5^
Xiao et al., 2011 [7]	Resistor on dielectric membrane	1–1,000
Takashima et al., 2008 [31]	Resistor on dielectric membrane	0.002–10^5^
Jeon et al., 2016 [32]	Resistor on dielectric membrane	0.013–10^5^
Paul and Baltes, 1995 [33]	Resistor on dielectric membrane	100–10^6^
Wenzel and Bak, 1998 [34]	Resistor on diaphragm	10–10^5^
Qiu et al., 2009 [35]	Metallic wire	1–100
Brun et al., 2012 [36]	Silicon nanowire	50–10^5^
Ghouila-Houri et al., 2017 [37]	Microwire	10,000–8 × 10^5^
Schelcher et al., 2011 [38]	Ni-microbeam	3.3–10^5^
Wang et al., 2010 [39]	Microplate	0.1–10^5^
Santagata et al., 2011 [40]	Tube-shaped	0.133–1.33 × 10^5^
Mercier et al., 2012 [41]	Cr/Au-resistor on LiNbO_3_-substrate (SAW device)	0.001–10^5^
